# Interaction Between Macro‐ and Micro-Nutrients in Plants

**DOI:** 10.3389/fpls.2021.665583

**Published:** 2021-05-10

**Authors:** Suresh Kumar, Santosh Kumar, Trilochan Mohapatra

**Affiliations:** ^1^Division of Biochemistry, ICAR-Indian Agricultural Research Institute, New Delhi, India; ^2^Decode Genomics Private Limited, New Delhi, India; ^3^Indian Council of Agricultural Research, New Delhi, India

**Keywords:** nutrient homeostasis, nutrient interaction, nutrient pathways interaction, phosphorus, sulfur, iron, zinc, P-Fe-Zn tripartite interaction

## Abstract

Nitrogen (N), phosphorus (P), sulfur (S), zinc (Zn), and iron (Fe) are some of the vital nutrients required for optimum growth, development, and productivity of plants. The deficiency of any of these nutrients may lead to defects in plant growth and decreased productivity. Plant responses to the deficiency of N, P, S, Fe, or Zn have been studied mainly as a separate event, and only a few reports discuss the molecular basis of biological interaction among the nutrients. Macro-nutrients like N, P, and/or S not only show the interacting pathways for each other but also affect micro-nutrient pathways. Limited reports are available on the investigation of two-by-two or multi-level nutrient interactions in plants. Such studies on the nutrient interaction pathways suggest that an MYB-like transcription factor, phosphate starvation response 1 (PHR1), acts as a master regulator of N, P, S, Fe, and Zn homeostasis. Similarly, light-responsive transcription factors were identified to be involved in modulating nutrient responses in *Arabidopsis*. This review focuses on the recent advances in our understanding of how plants coordinate the acquisition, transport, signaling, and interacting pathways for N, P, S, Fe, and Zn nutrition at the molecular level. Identification of the important candidate genes for interactions between N, P, S, Fe, and/or Zn metabolic pathways might be useful for the breeders to improve nutrient use efficiency and yield/quality of crop plants. Integrated studies on pathways interactions/cross-talks between macro‐ and micro-nutrients in the agronomically important crop plants would be essential for sustainable agriculture around the globe, particularly under the changing climatic conditions.

## Introduction

Plant growth and development are largely determined by nutrient availability; therefore to ensure better productivity of crop plants, it becomes essential to understand the dynamics of nutrients uptake, transport, assimilation, and their biological interactions (Wawrzyńska and Sirko, 2014). A wealth of information has been generated during the last two decades on morphological and physiological adaptations of plants in response to the changes in the availability of mineral nutrients (Gniazdowska and Rychter, 2000; Maathuis, 2009; Krouk et al., 2011; Gruber et al., 2013; Zhao and Wu, 2017; Krouk and Kiba, 2020). Protein-coding genes involved in the uptake, mobilization, storage, and assimilation of macro/micro-elements have been characterized to some extent; and regulatory networks affecting their expression in response to the changing nutritional status are being elucidated (Schachtman and Shin, 2007; Giehl et al., 2009; Gojon et al., 2009; Liu et al., 2009; Pilon et al., 2009; Hindt and Guerinot, 2012; Vigani et al., 2013; Briat et al., 2015; Chaiwong et al., 2020). Crop plants are frequently subjected to nutrients imbalance which adversely affects several metabolic processes. However, plants have evolved strategies to cope up with nutritional deficiencies. Although a large number of elements are naturally available in the soil, 17 elements are currently known to be important for the proper growth and development of crop plants. While Nitrogen (N), Phosphorus (P), Potash (K), Calcium, Sulfur (S), and Magnesium are known as macro-nutrients (required in comparatively larger amounts), Iron (Fe), Zinc (Zn), Copper, Boron, Manganese Molybdenum, Chloride, and others are the micro-nutrients (required in a smaller quantity) for the growth and development of crop plants. The use of N and P fertilizers has been one of the key factors to produce enough food materials to feed the burgeoning human population world over, which is considered to be one of the important components of the *Green Revolution* during the 1960s (Kumar, 2013).

Nitrogen is one of the nutrients essentially required for the vegetative growth of crop plants as it is needed for the synthesis of starch in leaf, production of amino acids for protein synthesis, and thus yield of the crop. Phosphorus is an essential constituent of nucleic acids, cellular membranes, and enzymes. It is needed for diverse cellular processes like photosynthesis, carbohydrate metabolism, energy production, redox-homeostasis, and signaling. P works as an activator for more than 60 enzymes in plants, regulates water content, and reduces the adverse effects of salts in plants. Similarly, sulfur is essentially required for the synthesis of amino acids like cysteine and methionine, as a cofactor/prosthetic group in Fe-S center, thiamine, S-adenosyl methionine, and in several primary and secondary metabolites (Wirtz and Hell, 2006; Khan et al., 2010; Koprivova and Kopriva, 2014). The majority of S in living organisms is present in a reduced form of organic-sulfur and thiols, while it is predominantly present in the oxidized-inorganic forms in the environment. Only plants, algae, fungi, and bacteria are capable of S assimilation (taking up the inorganic-sulfate from the soil, reducing it to sulfide and synthesize various biomolecules; Davies et al., 1996; Maruyama-Nakashita et al., 2004; Koprivova and Kopriva, 2014). Deficiency symptoms of S resemble those of N-deficiency: the leaves become pale-yellow. However, unlike nitrogen deficiency, the symptoms appear first on the younger leaves and persist even after an adequate supply of nitrogen. Moreover, S availability in the soil fluctuates; hence, a plant needs to reprogram its metabolism according to the changing nutrients status.

The micro-nutrients like Fe and Zn play very important roles in the physiological processes of crop plants; however, they are required in very little amounts. Fe is required for chlorophyll synthesis and maintenance of chloroplast structure and functions. It is generally present in higher quantities in soil, but its bioavailability becomes limited in aerobic and neutral pH environments (Colombo et al., 2014). In aerobic soils, Fe is found predominantly in the Fe^+3^ form, with extremely low solubility, which does not fulfills the plant’s iron requirement. Hence, Fe-deficiency becomes a common nutritional disorder in many crop plants, resulting in interveinal-chlorosis in young leaves, stunted root growth, poor yield, and reduced nutritional quality. Similarly, Zn is required for optimum plant growth, as it influences several biological processes including cell proliferation, carbohydrate metabolism, and P-Zn interactions (Rehman et al., 2012). Zn is the only metal required for all the six classes (hydrolases, oxidoreductases, lyases, transferases, ligases, and isomerases) of enzymes (Coleman, 1998). Although it plays a structural role in some of the regulatory proteins (Berg and Shi, 1996), its higher concentration is toxic for the cell (Sresty and Madhava Rao, 1999; Xu et al., 2013). Zn deficiency in a plant results in deformed chlorotic leaves, interveinal necrosis, decreased photosynthesis, and reduced biomass production leading to reduced plant growth, lesser yield, and poor nutritional quality of the produce (Zhao and Wu, 2017).

Reports on interactions between multiple nutrient elements suggest that they affect uptake, transport, or assimilation of each other. Therefore, multi-level interactions between the nutrient elements need to be studied to better understand the sensing and signaling pathways triggered in response to the varying availability of nutrient elements. The multi-level study, integrating the transcriptome through enzymatic activities to the metabolome, helps to understand the strategies of a plant to reprogram metabolic pathways in response to the deficiency, resupply, sufficiency, and/or excessiveness of mineral nutrients. This provides insights into how plants adjust metabolic pathways in the absence/resupply of mineral nutrient(s) for its proper growth and development (Amtmann and Armengaud, 2009; Kellermeier et al., 2014). Phosphorus and sulfur being essential macro-nutrients for plant growth, development, and productivity, they show interactions in terms of substituting phospholipids with sulfolipids and galactolipids in cellular membranes under P-deficiency stress (Okazaki et al., 2013). While such biological interactions between N, P, and S are well-known (Aulakh and Pasricha, 1977; Sinclair et al., 1997; Smith et al., 2000; Gojon et al., 2009; Islam et al., 2012; Chotchutima et al., 2016; Krouk and Kiba, 2020), the knowledge of signaling pathways involved in responses to nutrient availability/deficiency is still limited. Nutritional deficiencies and interactions are not restricted to macro-elements only, micro-nutrients, such as Zn and Fe, have their homeostasis and show biological interactions. The interactions between Zn and Fe have also been studied (Connolly et al., 2002; Kerkeb et al., 2008; Barberon et al., 2011; Haydon et al., 2012; Xie et al., 2019). Deficiency of micro-element results in certain physiological disorders impacting plant growth, development, and productivity. Such interactions have been partially understood at physiological and molecular levels, the intricate nutritional cross-talks need to be extensively studied to maximize crop productivity.

## Interaction Between N and P Homeostasis in Plants

**Figure 1:**
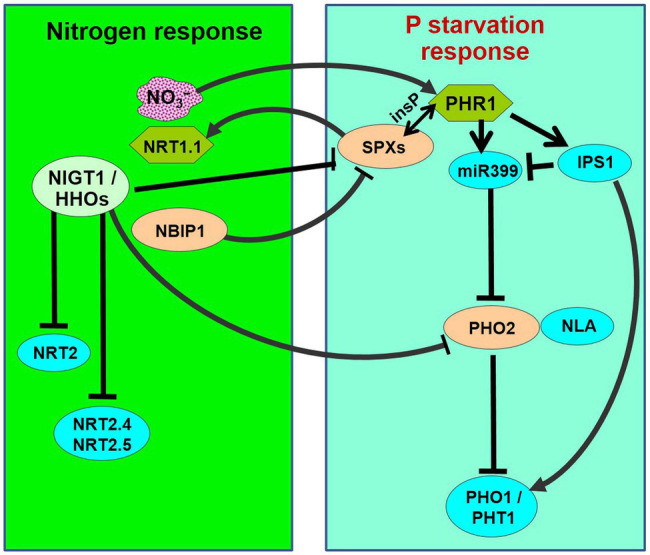
Schematic representation of nitrogen-starvation response and phosphate-starvation responses explaining N/P interactions. PHR1 acts as a major transcriptional regulator of P-starvation response, which is accompanied by the activation of phosphate starvation-induced (PSI) genes followed by phosphate uptake and translocation by phosphate transporters (PHO1 and PHT1s). PHR1 is negatively regulated by SPXs through inositol polyphosphate (insP)-triggered Pathway. During P-starvation, PHR1 up-regulates the IPS1 and miR399 expression. miR399 represses PHO2, which acts in association with NLA (an E3 ligase) to repress/degrade PHO1 and PHT1. OsSPX4 is degraded through 26S proteasome pathway in response to N supply (+N) *via* the action of OsNRT1.1B and an E3 ligase OsNBIP1 (NRT1−NBIP). SPXs transcription is directly repressed in response to +N by NIGT1/HHOs. On the contrary, PHR is positively regulated by +N. PHO2 expression is down-regulated in response to +N by NIGT1/HHOs and CHL1/NRT1.1. Thus, the phosphate-starvation response is attenuated by N-starvation because of the accumulation of negative regulators (SPXs and PHO2) and a decrease in the positive regulator (PHR1).

Owing to the Haber-Bosch process, N availability is considered to be virtually infinite but the global P reserves are becoming scarce for agriculture in the 21st century. Therefore, understanding plant responses to the availability of these nutrients and biological interactions are crucial for reduced/optimum fertilizer use in agriculture (Medici et al., 2019). The effects of N‐ and P-fertilizer on crop yield have been largely studied in isolation, but recent findings suggest interactions between the macro-nutrients. Elser et al. (2007) reported synergistic interactions between N and P in providing a much higher yield under diverse ecosystems. While an adequate supply of N positively affects the uptake of P (Smith and Jackson, 1987), P-starvation negatively affects N uptake and assimilation (Gniazdowska and Rychter, 2000). This suggests a mutual interaction between N and P nutrition in plants (Güsewell, 2004). For a crop plant to successfully reach its reproductive phase, sufficient availability of the essential mineral nutrients, such as N, P, and K, needs to be ensured for various biochemical, physiological, and metabolic processes to occur appropriately. N is not only required as a nutrient for the synthesis of starch and amino acids, but nitrate (N) also acts as a signal molecule to modulate phosphate response, and to coordinate the N–P balance (Figure 1).

N-related long-distance signaling involves cytokinin biosynthesis, C-terminal encoded peptide (CEP), and glutaredoxins (Tabata et al., 2014; Ohkubo et al., 2017; Poitout et al., 2018). Interaction between N and P signaling was reported to be mediated by Nitrogen limitation adaptation (NLA) and *PHO2* that control phosphate transporter activity resulting in N-dependent P accumulation in shoots (Peng et al., 2007; Lin et al., 2013). A transcription factor GARP and Nitrate-inducible GARP-type translational repressor 1.4 (NIGT1.4) were reported to affect primary root growth according to the nitrate and phosphate ion signals *via* transcription and protein accumulation, respectively (Medici et al., 2015). Moreover, PHR1 was reported to be a central regulator of NIGT1 (Kiba et al., 2018). Cerutti and Delatorre (2013) reported N–P interaction in modulating root architecture by a regulatory component (PDR1) of N and P signaling mediated by cytokinin. While P-starvation triggers the formation of shorter primary and lateral roots (Hufnagel et al., 2014; Zhang et al., 2019) to better explore the soil for P acquisition, N-deficiency represses lateral root development and favors primary root elongation to explore deeper soil for better N acquisition (Jia and von Wiren, 2020). Such biological interactions might be the strategy of plants to coordinate N and P acquisition under varying nutritional conditions for optimum growth and yield. However, our current understanding of the molecular basis of such interaction is still elusive.

Thus, evidence suggests that N availability modulates phosphorus starvation responses (Rufty et al., 1990; Kant et al., 2011; Liang et al., 2015; Medici et al., 2019). Under P-starvation, N supplementation activates P acquisition, while N-starvation represses the P-starvation responses. This indicates that a plant modulates its regulatory system to prioritize N nutrition over P. Three major signaling factors involved in N–P interaction have been identified, which include SPXs, PHRs, and PHO2. Expression of SPX1, SPX2, and SPX4 was reported to be repressed in response to N supplementation in *Arabidopsis* and rice (Kiba et al., 2018; Ueda et al., 2020). In rice, a nitrate sensor [nitrate transporter (NRT), NRT1.1B] was reported to interact with a phosphate-signaling repressor (SPX4; Hu et al., 2019). Phosphate starvation response (PHR) was reported to be positively regulated by N at transcriptional and post-transcriptional levels (Sun et al., 2018; Varala et al., 2018). However, PHR1 stability was reported to decrease by N-starvation (Medici et al., 2019). Moreover, the microRNA (miR827)–NLA module was reported to be involved in nitrate-dependent phosphate homeostasis in *Arabidopsis* (Kant et al., 2011). Evidence indicates an important role of cytokine in P and N signaling (Cerutti and Delatorre, 2013; Poitout et al., 2018). Several other potential factors involved in N-dependent PHR regulation have been reported in *Arabidopsis*, including miR399 (Liang et al., 2015) and NPF7.3/NRT1.5 (Cui et al., 2019); however, their roles in N-dependent PHR regulation are yet unexplored. Only a few proteins have been reported to be involved in nitrate transport in rice. Medici et al. (2019) reported that N-deficiency in rice strongly affects shoot growth, but N availability minimizes the effects of P-deficiency on shoot growth. More importantly, the effect of N-deficiency is less important for plants under P-deficiency. Recently, Pueyo et al. (2021) reported structural and functional modifications in roots, leading to the formation of clusters and altered nodule metabolism, under P and N deficiencies. Signaling factors, including phytohormones and miRNAs, were reported to be the important players in the N and P interactions. We observed down-regulated expression of a high-affinity nitrate transporter (LOC_Os02g38230) in roots of rice under P-starvation stress, with more down-regulation in the P-deficiency stress-sensitive (Pusa-44) rice genotype (our unpublished data). Hence, a comprehensive understanding of the interactions between the macro-nutrients would be essential to optimize/maximize the crop yields under diverse nutritional status in the soils.

## Interaction Between P and S Homeostasis in Plants

A major source of S for plants is the inorganic sulfate (SO_4_^−2^; Leustek et al., 2000), and several S transporters (SULTRs) have been functionally characterized in plants (Takahashi, 2010). It has also been reported that plant cells rapidly replace sulfolipids with phospholipids under S-deficiency, and phospholipids with sulfolipids during P-deficiency (Yu et al., 2002; Sugimoto et al., 2007; our unpublished data). Interestingly, the genes involved in the replacement of phospholipids with sulfolipids under P-deficiency in plants (*SQD1* and *SQD2*) contain a PHR1 binding sequence (P1BS) in the promoter and get induced by P-deficiency (Franco-Zorrilla et al., 2004; Stefanovic et al., 2007). Increased synthesis of miR395 was reported due to P-starvation (Hsieh et al., 2009), which increases S translocation from root to shoot by SULTR2;1, and enhances sulfolipid biosynthesis. Evidence for co-regulation of P-S signaling is getting accumulated. In *Arabidopsis*, the central regulator of P-starvation signaling (PHR1) is potentially involved in S, Fe, and Zn homeostasis as it regulates the expression of three sulfate transporters (SULT1;3, SULT2;1, and SULT3;4), two zinc transporters (Zinc/Iron-regulated transporter (Zrt/Irt)-related proteins, ZIP2 and ZIP4), and a ferritin (FER1) protein (Rouached et al., 2011; Bournier et al., 2013; Bouain et al., 2014a; Briat et al., 2015). This suggests that macro‐ and micro-nutrient homeostasis, at least partially, relies on the regulation of the expression of transporter genes. We observed up-regulated expression of two sulfate transporters (LOC_Os01g52130 and LOC_Os06g05160) in rice, with higher expression in P-deficiency tolerant genotype, under P-starvation. Such nutrients’ homeostasis is supposed to make NIL-23 to be tolerant to P-deficiency stress. PHR1 has been reported to positively regulate SULTR1;3 expression, while it negatively affects the expression of SULTR2;1 and SULTR3;4 under P-deficiency (Rouached et al., 2011).

Recently, Garcia et al. (2021) reported the involvement of phytohormone ethylene (ET) in the regulation of crosstalk between P, S, or Fe deficiency. Some of the key elements of the ET transduction pathway (CTR1, EIN2, and EIN3/EIL1) were reported to play roles in nutrient deficiency responses. Dicot plants, like *Arabidopsis*, adopt several strategies (mainly in roots) to facilitate mobilization/uptake of nutrients to cope up with P, S, or Fe deficiency. Such responses include modification in root morphology, increased activity of transporters, enhanced synthesis/release of nutrient solubilizing compounds, and improved activities of ferric reductase or phosphatase activity.

## Interactions Between P, Fe, and Zn Homeostasis in Plants

Cross-talks between P, Zn, and Fe homeostasis have been reported earlier in many plants (Briat et al., 2015), molecular basis and biological significance of the nutritional interactions have largely been unknown. Complex tripartite cross-talks among P, Zn, and Fe are being reported (Zheng et al., 2009; Briat et al., 2015; Rai et al., 2015; Xie et al., 2019). Whole transcriptome analysis revealed more than 500 overlapping genes regulated by both P‐ and Fe-deficiency in roots of rice and *Arabidopsis* (Zheng et al., 2009; Li and Lan, 2015). Gene expression in plants under P-deficiency/starvation is determined by the presence/absence of Fe (Misson et al., 2005; Thibaud et al., 2010). Fe-deficiency was reported to alter the transcription of P assimilation-related genes (Zheng et al., 2009; Moran et al., 2014). Under P-starvation, expression of *FER1* (encoding Fe storage protein ferritin), *NAS3*, and *YSL8* genes (responsible for Fe homeostasis) are induced (Bustos et al., 2010; Bournier et al., 2013). In *Arabidopsis*, double mutations for *phr1 phl1* altered Fe distribution and expression of Fe-related genes (Bournier et al., 2013; Briat et al., 2015). This suggests that PHR1 and PHL1 might be involved in integrating P and Fe nutrient signaling. A high-affinity copper (Cu) transporter COPT2 was reported to act as a key player in the interaction between P‐ and Fe-deficiency signaling in *Arabidopsis* (Perea et al., 2013). COPT2 plays a dual role under Fe-deficiency; it helps in Cu uptake and distribution to minimize Fe losses (Xie et al., 2019). Moreover, loss of functions of COPT2 aggravates the P-starvation responses in *Arabidopsis*. We observed up-regulated expression of Fe^2+^ transporter genes in roots and shoots of the tolerant rice (NIL-23) genotype, whereas down-regulated expression of the transporters was observed in the sensitive (Pusa-44) genotype under P-starvation stress. Notably, a rice vacuolar-membrane transporter OsVIT1 (LOC_Os09g23300) was observed to be induced by ~1.5-fold in roots and shoots of NIL-23 under the stress, while it was significantly down-regulated (>2.5-fold) in roots and shoots of Pusa-44 (a stress-sensitive rice genotype).

**Figure 2:**
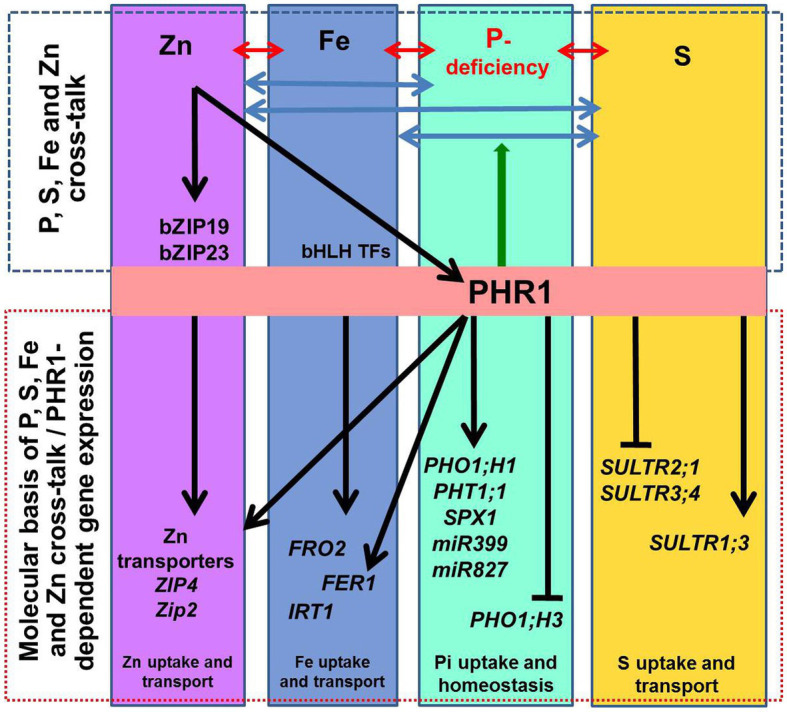
Schematic representation of the macro‐ and micro-nutrient homeostasis. The interactions between phosphorus (P), iron (Fe), sulfur (S), and zinc (Zn) homeostasis are indicated by ↔ arrows. At the molecular level, transcription factor PHR1 (initially identified as a key regulator of the phosphate-starvation induced genes) up-regulates phosphate transporters (*PHT1;1*), *PHO1;H1*, and the genes involved in phosphate deficiency sensing/signaling (*SPX1*, *miR399*, and *miR827*). Other genes known to be involved in P-deficiency signaling/sensing include *miR827* and *miR399*. Transcriptional regulation of some of the genes involved in maintaining Fe and Zn homeostasis is PHR1-dependent; it includes *FER1* (encoding Fe storage protein ferritin) and *ZIP2* and *ZIP4* (zinc transporters). PHR1 also acts as a central regulator of sulfate transport (*SULTR1;3*, *SULTR2;1*, and *SULTR3;4*). The arrow-heads and flat-ended lines indicate the positive and negative effects of PHR1, respectively. PHR1 acts as a regulator of P-transporters (PHT1 and PHO1) *via* the PHR1–miR399–PHO2 module. Also, *ZIP2* and *ZIP4* are activated by PHR1 binding to the P1BS sequences in the promoter of the genes. Likewise, Zn sufficiency inactivates the Zn-regulatory network and represses Zn transporters for Zn homeostasis. Besides, *PHO1;H3* is repressed by sufficient Zn supply, and the PHR1 and PHO1 proteins help to maintain the Pi–Zn homeostasis cross-talk. Similarly, Fe homeostasis is also regulated in a PHR1-dependent manner.

Similarly, Zn-deficiency induces the expression of several P assimilation-related genes (van de Mortel et al., 2006), while P-deficiency activates the expression of the genes involved in Zn and Fe homeostasis (Misson et al., 2005; Bustos et al., 2010). Expression of several *ZIP* genes (*OsZIP1*, *OsZIP4*, *OsZIP5*, plasma membrane Zn transporters) was reported to be induced by Zn-deficiency and controlled by the availability of divalent cations such as Zn^2+^, Fe^2+^, Cu^2+^, Mn^2+^ in rice (Suzuki et al., 2012). We observed up-regulated expression of *OsZIP3* and *OsZIP4* (LOC_Os04g52310 and LOC_Os08g10630) in NIL-23, while they were down-regulated in Pusa-44 under P-starvation stress. Fe-deficiency caused up-regulated expression of the genes involved in Zn uptake and homeostasis in leaf and root of soybean (Moran et al., 2014). A Fe-deficiency-responsive gene *AtIRT1* was reported to play a key role in coordinating the signaling for Zn‐ and Fe-deficiency in *Arabidopsis* (Briat et al., 2015). An MYB family transcription factor PHR1 acts as a common regulator of P, Fe, and Zn homeostasis, and functions as an integrator of multiple nutrient signals (Briat et al., 2015). Transcriptional activation of some of the genes involved in Fe homeostasis was reported to be PHR1-dependent (Figure 2), including *FER1* (encoding the Fe storage protein), and *PHO1;1* (encoding P transporter). PHO1;1 was reported to be involved in coordination between Fe transport and P–Zn deficiency signaling in rice (Saenchai et al., 2016). Excessive Zn was reported to cause Fe deficiency because of decreased IRT1 protein in *Arabidopsis* root (Connolly et al., 2002) due to ubiquitin-mediated proteasomal degradation of IRT1 (Kerkeb et al., 2008; Barberon et al., 2011). Moreover, Fe starvation was also reported to affect S uptake and assimilation. Forieri et al. (2013) reported 2.5-fold down-regulation of high-affinity S transporter SULTR1;1 under Fe deficiency. The role and abundance of Fe–S cluster in various nutritional stresses need to be studied (Forieri et al., 2013). However, the basics of the cross-talk between P-, Fe-, Zn and S-deficiency signaling in plants remain to be elucidated. We also observed that P-starvation stress increased carbon-flux *via* glycolysis for the synthesis of organic acids, altered lipid metabolism, and Fe/Zn metabolism, which corroborate with the earlier report (Wasaki et al., 2003).

Leskova et al. (2017) reported enhanced uptake of Zn to mimic Fe-deficiency by high ferric-chelate reductase activity, not due to Zn-inhibited Fe uptake but Zn-simulated transcriptional response of Fe-regulated genes. This indicates that Zn affects Fe homeostasis by sensing the availability of Fe. Recently, Brumbarova and Ivanov (2019) reported the involvement of three transcriptional regulators (HY5, PIF4, and the NF-Y complex) in modulating nutrient responses in *Arabidopsis*. These transcriptional regulators play important role in light signaling and modulate global transcriptome to adjust nutrient availability.

## Master Regulators of Multiple-Nutrient Homeostasis

Transcription factor PHR1 was initially identified as a major regulator of P homeostasis in plants (Fujii et al., 2005; Aung et al., 2006; Bari et al., 2006). Subsequently, reports indicate that PHR1 also regulates the expression of genes involved in S, Fe, and Zn homeostasis (Rouached et al., 2011; Bournier et al., 2013; Khan et al., 2014). Thus, PHR1 is considered as a molecular link between the pathways controlling macro‐ and micro-nutrient homeostasis. The regulatory role of PHR1 has been documented based on two-by-two interactions of some of the nutrient elements like P and S, P and Fe, P and Zn. PHR1 is one of the important regulators of P-deficiency responses, but other regulators like WRKY45/75, ZAT6, MYB62, PTF1, and bHLH32 have also been reported.

Subsequently, higher-order coordinators such as light-response transcription factors/complexes (PIF4, HY5, and the NF-Y) were identified as master transcriptional regulators coordinating plant growth and nutrient utilization (Brumbarova and Ivanov, 2019). Based on the available reports and nutritional interactions, it can be concluded that PHR1 and HY5 act as master regulators of multiple nutrient homeostasis. Moreover, the role of miRNAs as a potential regulator of the cross-talks between the nutrient homeostasis is also being deciphered (Hsieh et al., 2009; Pant et al., 2009; Liang et al., 2015; Pueyo et al., 2021). The involvement of epigenetic and epitranscriptomic marks (Wang et al., 2016; Kumar et al., 2018; Kumar and Mohapatra, 2021) in regulating nutrient interactions is yet to be explored. The need of the day is to conduct more extensive, multi-level interaction studies with a system biology approach, and to decipher the integrative gene-networks to better manage the nutrient deficiencies in crop plants, towards maximizing the yield and quality of the produce (Kumar, 2018).

## Conclusion

The combinations of high-throughput “*Omics*” and reverse genetics approaches have resulted in the characterization of genes involved in the interactions between multiple nutrients homeostasis. Interactions between macro-nutrients have been evident from the morphological, physiological, and agronomic studies; however, molecular bases of such biological interactions between macro-, micro-, and macro-micro-nutrients are being elucidated. Biological interactions have not only been detected between N and P, but P and micro-nutrients (Fe and Zn) have also been reported in plants (Bouain et al., 2014b; Briat et al., 2015), which would be very important to maximize crop yield, particularly on marginal lands under the changing climatic conditions (Kumar, 2020). Studies involving various combinations of macro‐ and micro-nutrient stress, and integrative signaling molecules would provide the genetic-basis for multi-partite cross-talks in plants. Therefore, future research would also need to focus on integrative studies to decipher the mechanisms involved in coordinating multiple nutrient interactions and nutrient-stress signaling to mitigate the harmful effects of nutrient(s) deficiency in crop plants. Besides, identification of the genes involved in the interactions between different nutrients (e.g., N, P, Fe, Zn, and/or Fe), their transport, and signaling in crop plants will help breeders/agronomists to develop alternate strategies for nutrient management in crops (Xie et al., 2019). In conclusion, the multi-partite integrative studies on the interactions between nutrient metabolic pathways would be of great importance for sustainable agricultural production/development all over the world (Kaur and Kumar, 2020).

## Author Contributions

SuK and TM conceived the idea. SuK supervised the experiments, data collection, and analyses. SaK performed bioinformatics analysis of data and prepared the draft manuscript. SuK, SaK, and TM revised the manuscript. All authors contributed to the article and approved the submitted version.

### Conflict of Interest

SaK is employed by Decode Genomics Private Limited, New Delhi, India.

The remaining authors declare that the research was conducted in the absence of any commercial or financial relationships that could be construed as a potential conflict of interest.
